# The efficacy of a multistrain probiotic on cognitive function and risk of falls in patients with cirrhosis: A protocol for systematic review and meta-analysis

**DOI:** 10.1097/MD.0000000000025535

**Published:** 2021-04-23

**Authors:** Na Wang, Wei Yao, Ruiping Ma, Fangfang Ren

**Affiliations:** a Department of Liver Disease; b Department of Urinary Transplantation; c Department of Hepatobiliary Surgery, The First Affiliated Hospital of Shandong First Medical University & Shandong Provincial Qianfoshan Hospital, Shandong, 250014, China.

**Keywords:** cognitive function, fall, meta-analysis, multistrain probiotic

## Abstract

**Objective:**

The effect of probiotics on cognitive function and the risk of falling in cirrhosis patients have not been previously evaluated. We perform this protocol for systematic review and meta-analysis to evaluate the effect of a multistrain probiotic on cognitive function and the risk of falls in patients with cirrhosis.

**Methods:**

An all-round retrieval will be performed in 5 electronic journal databases from their inception to March 2021, which comprise Medline, Pubmed, Embase, ScienceDirect, and the Cochrane Library by 2 independent reviewers. Data extraction was performed independently, and any conflict was resolved before final analysis. Only randomized clinical trials were included in this study. The main endpoints were cognitive function and risk of falls, and the secondary endpoints were fall incidence, health-related quality of life (HRQOL), systemic inflammatory response, gut barrier, bacterial translocation, and fecal microbiota. The risk of bias assessment of the included studies was performed by 2 authors independently using the tool recommended in the Cochrane Handbook for Systematic Reviews of Interventions.

**Results:**

We hypothesized that the multistrain probiotic improved cognitive function, risk of falls, and inflammatory response in patients with cirrhosis and cognitive dysfunction.

**Conclusion:**

This study expects to provide credible and scientific clinical evidence for the efficacy and safety of a multistrain probiotic on cognitive function and the risk of falls in patients with cirrhosis.

**OSF registration number:**

10.17605/OSF.IO/JKMTP.

## 1 Introduction

Cirrhosis is a diffuse process of liver damage considered irreversible in its advanced stages.^[1,2]^ In 2016, more than 40,000 Americans died because of complications related to cirrhosis, making it the 12th leading cause of death in the United States.^[3]^ Recent projections suggest that this number is likely to grow. An estimated 630,000 Americans have cirrhosis, yet less than 1 in 3 knows it. Cirrhosis and advanced liver disease cost the United States between $12 billion and $23 billion dollars in health care expenses annually.^[4]^

Intestinal dysbiosis, gut barrier failure, bacterial translocation, and subsequent inflammatory response are key factors in the progression of cirrhosis.^[5–7]^ These factors have also been implicated in worsening of liver failure and portal hypertension and, consequently, in the development of complications, including cognitive dysfunction, after advanced chronic liver disease, or cirrhosis is established. Cognitive dysfunction is a risk factor for overt hepatic encephalopathy, worsening in health-related quality of life (HRQOL), traffic accidents, and mortality in patients with cirrhosis.^[8,9]^ In addition, these patients are more predisposed to accidental falls.

Falls are particularly important in patients with cirrhosis, because they have a greater risk of fracture than the general population.^[10,11]^ Moreover, falls are a significant cause of complications, mortality, and HRQOL impairment. In addition to their negative consequences for the patient, falls have implications for the patient's relatives and are an economic and social burden for the community. Moreover, individuals with previous falls are frequently predisposed to recurrent falling, thus supporting the growing concept of frailty in patients with cirrhosis and the need for preventive measures.

Studies have shown that certain probiotics improve the proinflammatory state, liver function, portal hemodynamics, and HRQOL in patients with cirrhosis.^[12,13]^ However, the effect of probiotics on cognitive function and the risk of falling in these patients has not been previously evaluated. We perform this protocol for systematic review and meta-analysis to evaluate the effect of a multistrain probiotic on cognitive function and the risk of falls in patients with cirrhosis.

## 2 Methods

### 2.1 Study protocol and registration

This protocol was designed in accordance with Cochrane Handbook for Systematic Reviews of Interventions and Preferred Reporting Items for Systematic Reviews and Meta-Analyses Protocols (PRISMA-P) 2015 checklist.^[14]^ The prospective registration has been approved by the Open Science Framework (OSF) registries (https://osf.io/jkmtp), and the registration number is 10.17605/OSF.IO/JKMTP. Ethical approval is not necessary because this is a meta-analysis.

### 2.2 Eligibility criteria

#### 2.2.1 Types of included studies

Randomized controlled trials (RCTs) are eligible for inclusion which should assess at least 1 outcome. Quasi-RCTs, literature review, duplicated publications, case report, animal experiments, editorials, and pharmaceutical experiments will be excluded.

#### 2.2.2 Participants

Patients diagnosed as cirrhosis by any guidelines or expert consensus with clear diagnostic criteria will be included, regardless of age, region, race, or country.

#### 2.2.3 Interventions and comparisons

The probiotic formulation was a mixture of 8 strains, namely, Streptococcus thermophilus DSM 24731, Bifidobacterium breve (B. breve) DSM 24732, B. longum DSM 24736, B. infantis DSM 24737, Lactobacillus paracasei (L. paracasei) DSM 24733, L. acidophilus DSM 24735, L. delbrueckii subsp bulgaricus DSM 24734, and L. plantarum DSM 24730, at a total dose of 450 billion live bacteria per 4.4 g sachet with maltose and silicon dioxide as excipients. The placebo contained maltose and silicon dioxide as inactive agents and was formulated as identical in appearance to the active agent. Patients were instructed to keep the study product at 4°C in the refrigerator at home and to take 1 sachet diluted in 1 glass of water, milk, or juice at room temperature twice daily (every 12 hours) over 12 weeks.

#### 2.2.4 Outcomes

At baseline and at the end of treatment, we assessed cognitive function according to Psychometric Hepatic Encephalopathy Score (PHES) and the risk of falls by means of the Timed Up and Go test and gait speed. The main endpoints were cognitive function and risk of falls, and the secondary endpoints were fall incidence, HRQOL, systemic inflammatory response, gut barrier, bacterial translocation, and fecal microbiota.

### 2.3 Information sources

An all-round retrieval will be performed in 5 electronic journal databases from their inception to March 2021, which comprise Medline, Pubmed, Embase, ScienceDirect, and the Cochrane Library.

The following key words were used on combination with Boolean operators AND or OR: “cirrhosis” OR “cirrhotic” OR “hepatic fibrosis,” “probiotic OR microbial,” “fall” OR “fracture.” References of the included articles were also scanned for potentially relevant studies. No restrictions were placed on the publication language.

### 2.4 Studies selection

**Figure 1:**
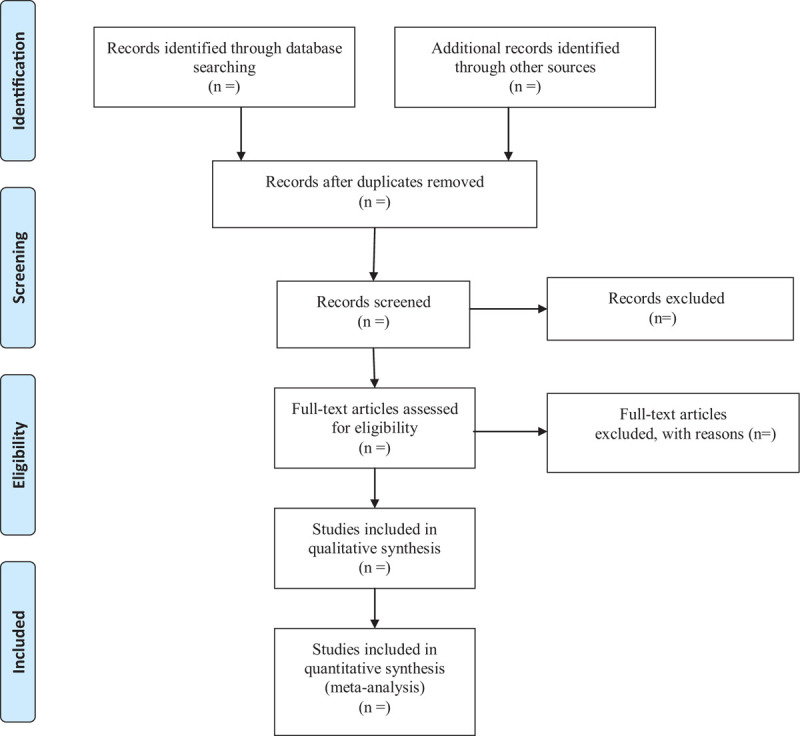
Flow diagram of study selection.

All retrieved literature will be imported into Note Express V3.0 software and duplicate literature will be eliminated. In the first instance, 2 researchers will independently and respectively screened the included studies on the basis of the inclusion criteria, and then download the remaining studies for further screening by reading full texts. If any disagreement arises in the process, it will be decided through discussion or by a third senior researcher. Two researchers will take notes of all excluded literature and provide reasonable reasons for exclusion. Details of the selection process will be presented in the PRISMA flow chart (Fig. 1).

### 2.5 Data extraction

The key information of the included trails will be extracted by 2 independent reviewers in accordance with the pre-designed form. The final decisions will be made by consensus process and disagreements will be adjudicated by a third high-level reviewer. We will collect the following information from each clinical trial included: the first author, publication year, primary locality of the study, sample size (research group/control group), outcomes, range of age (research group/control group), gender distribution (male/female), diagnostic criteria, and funding. If any material information elements are missing, we will attempt to contact the authors for the desired data. We will employ intention-to treat analysis in case of missing data are unobtainable. Sensitivity analysis will also be executed to address the potential impact of missing data, which will be discussed if necessary.

### 2.6 Risk of bias assessment

Two investigators will assess the methodological quality of the RCTs separately and individually by risk of bias 2.0 tool recommended by Cochrane handbook.^[15]^ This tool can be applied to evaluate the possibility and sources of bias in RCTs comprehensively from 5 areas, which are “risk of bias arising from the randomization process,” “risk of bias due to deviations from the intended interventions,” “missing outcome data,” “risk of bias in measurement of the outcome,” and “risk of bias in selection of the reported result.” For several signaling questions set up by each module, the investigators should respond to “Yes,” “Probably yes,” “Probably no,” “No (not),” “Not applicable,” or “No information (unclear),” respectively. If any divergence arises during this procedure, the 2 investigators will reach agreement through discussion or be adjudicated by a third senior investigator.

### 2.7 Assessment of quality of evidence

Grading of recommendations assessment, development, and evaluation (GRADE) system will be adopted to assign levels for meta-analysis evidence (Table 1).^[16]^ The confidence of evidence pooled within the systematic review and meta-analysis will be appraised independently and separately by 2 review authors. GRADE is not only a rating system for proofs, but also summarizes evidence for systematic reviews and guidelines in the field of health care and provides a transparent, reliable, and structured method. RCT is initially identified as high-quality proof. Study limitations/risk of bias, publication bias, imprecision, inconsistency, and indirectness are 5 factors that may degrade the confidence of evidence quality. The overall quality of evidence will be defined as “high,” “moderate,” “low,” or “very low.” Any disagreement during this process will be reached by 2 investigators through consultation or adjudicated by a third senior investigator.

**Table 1:** Quality of the evidence and recommendation strengths.

Quality assessment							
Number of RCT	Limitations	Inconsistency	Indirectness	Imprecision	Outcome measures	Quality	Importance
Cognitive function							
							
Risk of falls							
							
Fall incidence							
							
Health-related quality of life							
							
Systemic inflammatory response							
							
Gut barrier							
							
Bacterial translocation							
							
Fecal microbiota							
							

### 2.8 Statistical analysis

We will perform meta-analysis using Stata 13.1 software (Stata-Corp LP, College Station, TX). If available data are insufficient, a descriptive analysis will be carried out. The *Q* test and *I*^2^ values will be used to indicate inter-study heterogeneity. When the *P* value of *Q* test >.1 and *I*^2^ < 50%, a fixed-effects model was applied; otherwise, a random-effects model was used. Binary variables were expressed by odds ratio with 95% confidence interval, and continuous variables by mean difference with 95% confidence interval.^[17]^ If significant heterogeneity is found, we will try to explore the source of heterogeneity by subgroup analysis based on specified effect modifiers as follows: interventions, publication year, participant's average age, sample size, publication language, and so on.

## 3 Discussion

Previous studies have been limited in their ability to provide strong evidences, such as small sample size and inconsistent adherence to modern methodological research standards, making it difficult to draw meaningful conclusions from individual trials. Thus, in order to provide new evidence based medical evidence for clinical treatment, we undertook a meta-analysis to evaluate the effect of a multistrain probiotic on cognitive function and the risk of falls in patients with cirrhosis who also have cognitive dysfunction and/or previous falls. We also studied potential mediators of these effects, particularly the systemic inflammatory response, the gut barrier, bacterial translocation, and intestinal microbiota. Our review process will be very rigorous and this article is a protocol of the systematic review and meta-analysis, which presents the detailed description of review implement. The results of our review will be reported strictly following the PRISMA criteria and the review will add to the existing literature by showing compelling evidence and improved guidance in clinic settings.

## Author contributions

Fangfang Ren designs the protocol. Wei Yao and Ruiping Ma perform the data collection. Na Wang writes the manuscript. All of the authors approved the submission.

**Conceptualization:** Wei Yao.

**Investigation:** Ruiping Ma.

**Methodology:** Ruiping Ma.

**Writing – review & editing:** Na Wang, Fangfang Ren.
